# Domino effect of pituitary growth hormone tumor complicated by diabetic ketoacidosis and pituitary apoplexy: a case report

**DOI:** 10.1186/s12902-021-00768-9

**Published:** 2021-05-26

**Authors:** JinYu Pan, XiangHong Yang, Wei Zhu

**Affiliations:** 1 Graduate Department, BengBu Medical College, 233030 Bengbu, Anhui China; 2 Department of Intensive Care Uint, Zhejiang Provincial People’s Hospital (People’s Hospital of Hangzhou Medical College), 310014 Hangzhou, Zhejiang China; 3 Department of Emergency Medicine, Zhejiang Provincial People’s Hospital (People’s Hospital of Hangzhou Medical College), 158 Shangtang Road, 310014 Hangzhou, Zhejiang People’s Republic of China

**Keywords:** Case report, Acute pancreatitis, Diabetic ketoacidosis, Hypertriglyceridemia, Pituitary growth hormone tumor

## Abstract

**Background:**

Patients with growth hormone (GH)-secreting adenoma usually develop glucose intolerance. GH increases metabolic rate and, when secreted aberrantly, may result in metabolic syndrome. Herein, we examine the associations of pituitary tumor-induced secretion of hormone with insulin resistance and metabolic syndrome, and determine the relation of pituitary tumor apoplexy-induced diabetic ketoacidosis (DKA) and acute pancreatitis.

**Case presentation:**

A 44-year-old male with a history of hypertension presented to the emergency department of our hospital on February 14, 2019 with symptoms of headache, dizziness, and vomiting. Computed tomography of the head revealed pituitary tumor with bleeding. An ultrasound scan of the abdomen revealed fatty liver and acute pancreatitis. Further examination revealed the presence of DKA, hypertriglyceridemia, cortical hypofunction crisis and acute kidney injury. Surgical endoscopic resection of the pituitary tumor resection via the transsphenoidal approach was performed. The patient’s postoperative recovery was remarkable.

**Conclusions:**

Long-term growth hormone abnormality may trigger insulin resistance, leading to metabolic syndrome and impaired glucose and lipid metabolism. The pituitary adenoma apoplexy may also directly induce DKA, creating a domino effect, which further deteriorate the aberrant metabolism of glucose and lipids.

**Supplementary Information:**

The online version contains supplementary material available at 10.1186/s12902-021-00768-9.

## Background

Patients with growth hormone-secreting adenoma usually develop glucose intolerance [1]. Growth hormone (GH) also increases the rate of lipid metabolism, resulting in elevated production of ketone bodies. Coexistence of impaired glucose metabolism, hyperinsulinemia, hypertriglyceridemia (HTG), and hypertension is characteristic of metabolic syndrome (MetS), a cluster of five individual risk factors, including hyperglycemia, hypertriglyceridemia, hypertension, abnormal lipid metabolism and abdominal obesity [2]. Although the precise etiology of MetS remains unclear, insulin resistance is a key causative factor, the result of excess circulating fatty acids [2]. HTG may result in acute pancreatitis (AP), which has a global incidence rate of 15–20 % [3]. Together, MetS, hyperglycemia and hyperlipidemia trigger a series of adverse effects in the course of AP [4, 5]. Here, we report a patient with pituitary tumor apoplexy who complicated diabetic ketoacidosis (DKA) and AP.

## Case presentation

A male patient presented at our hospital for emergency treatment after experiencing headache, dizziness and vomiting for one day. The patient had a four-year history of hypertension and had been receiving irbesartan, a blood pressure medication, for control, but his blood pressure was not being monitored regularly. When admitted to the hospital, his consciousness was lucid, body temperature 37.5 ℃, pulse 128 beats/minute, 22 breaths/minute, systolic/diastolic blood pressure 82/55 mmHg; left side pupil 3 mm, right side pupil 4 mm, sensitive to light reflection; no other neurological signs; obvious epigastric tenderness; no other abnormalities in cardiopulmonary abdominal physical examination. Laboratory data showed normal pO_2_ and pCO_2_, abnormal blood gas results (pH 7.327, actual base excess − 5.1 mmol/L, standard base excess − 5.0 mmol/L, bicarbonate 19.9 mEq/L), Lac 1.6 mmol/L, white blood cell (WBC) count 8.64 (10^9/L), positive C-reactive protein (31.8 mg/mL), and significantly increased serum levels of triglycerides (68.07 mmol/L), glucose (14.89 mmol/L), amylase (279.00 U/L) and lipase (382.00 U/L), slightly elevated serum follicle stimulating hormone (19.04 IU/L), luteinizing hormone (5.87 IU/L), prolactin (0.05 ng/mL), progesterone < 0.10 ng/mL, testosterone (1.08 ng/mL), cortisol (16.70 ug/dL), corticotropin (10.00 pg/mL), calcium (2.18 mmol/L), serum creatinine (54.40 mmol/L), and growth hormone (28.70 ng/mL). We did not measure the level of IGF-1, for it is positively associated with the level of GH [6]. The glycated hemoglobin HbA1 (17.6 %) and HbA1c (15.1 %) were both remarkably higher than the upper limit of normal range. Urine ketones (++++), coagulation factors and hepatic enzymes were within normal limits. The insulin level and C peptide level were not measured. Laboratory values are described in detail in Table 1.

**Table 1:** Laboratory findings during hospitalization

Laboratory test	Results	Normal range
**Blood biochemistry**		
WBC	8.64*10^9/L	4.50–11.00*10^9/L
Lactate	1.60 mmol/L	0.50-1.00 mmol/L
Serum creatinine	54.40 mmol/L	58.00-110.00 mmol/L
Glucose	14.89 mmol/L	4.20–6.100 mmol/L
C-reactive protein	31.80 mg/mL	0.00–10.00 mg/m L
Triglycerides	68.07 mmol/L	0.34–1.70 mmol/L
Total cholesterol	21.64 mmol/L	3.11–5.96 mmol/L
Pancreatic amylase	348.00 U/L	0.00–53.00 U/L
Lipase	382.00 U/L	16.00–63.00 U/L
Amylase	279.00 U/L	30.00-110.00 U/L
Calcium	2.18 mmol/L	2.10–2.80 mmol/L
Bicarbonate	19.90 mEq/L	21.00–29.00 mEq/L
HbA1	17.60 %	6.30-9.00 %
HbA1c	15.10 %	3.60-6.00 %
**Hormonal data**		
Cortisol	16.70 ug/L	67.00-226.00 ug/L
Corticotropin	10.00 pg/mL	0.00–46.00 pg/mL
Follicle stimulating hormone	19.04 IU/L	0.90–12.00 IU/L
Luteinizing hormone	5.87 IU/L	0.60–12.10 IU/L
Estradiol	< 10.00 pg/mL	11.00–44.00 pg/mL
Prolactin	0.05 ng/mL	3.50–19.40 ng/mL
Testosterone	1.08 ng/mL	1.60–8.10 ng/mL
Progesterone	< 0.10 ng/mL	0.13–0.97 ng/mL
Growth hormone	28.70 ng/mL	0.06-5.00 ng/mL
**Urinary biochemistry**		
Urine amylase	3900.00 U/L	32.00-641.00 U/L
Urine ketone bodies	**++++**	**-**

**Fig. 1:**
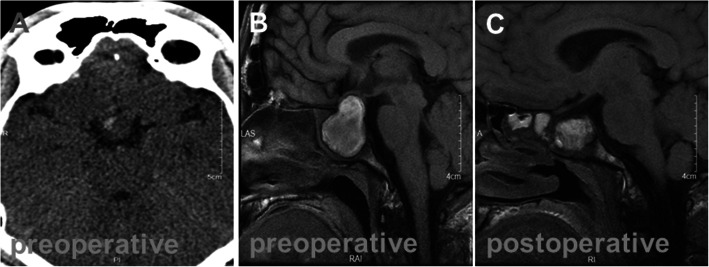
The brain images of the patient were evaluated by CT and MRI. **a** Preoperative computed tomography revealed pituitary hemorrhage (white arrow). **b** Preoperative contrast-enhanced T1-weighted magnetic resonance images exhibited pituitary tumor with hemorrhage, lobulated mass measuring 2.6 × 2.7 × 3.2 cm at the sella region with a prominent suprasellar component and vascular encasement. **c** Postoperative MRI images showed no residual tumor

**Fig. 2:**
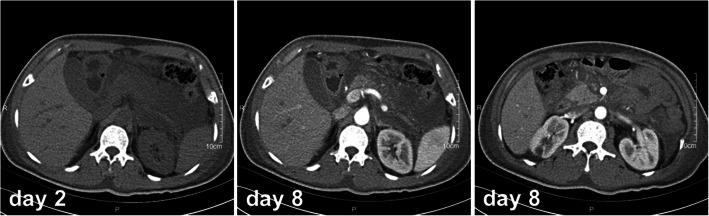
Abdominal computed tomography exhibited acute necrotizing pancreatitis with peripancreatic effusion and local intestinal wall thickening on day 2 and day 8 of admission

Relevant imaging examinations on admission revealed that computed tomography (CT) and magnetic resonance imaging (MRI) of the head indicated pituitary tumor with hemorrhage (Fig. 1) and acute necrotizing pancreatitis with peripancreatic effusion (Fig. 2). According to admitted results of above examinations and abdominal ultrasound, patients were diagnosed as severe acute pancreatitis, diabetic ketoacidosis, emergency pituitary tumor with hemorrhage, hypovolemic shock, fatty liver and hypertension. Diabetic ketoacidosis was diagnosed due to the abnormal results of blood gas test, which suggested the patient may have metabolic acidosis even though the pH value was not so much low. On the other hand, hypertension was diagnosed 4 years ago, which may indicate the time when GH excess started.

The patient’s symptoms of headache, vomiting, abdominal distention, shortness of breath and oliguria disappeared at 3 day of emergency intensive care unit (EICU). The patient was ill on February 14, 2019. He underwent transsphenoidal endoscopic pituitary tumor resection 11 days after being transferred from EICU. His postoperative recovery was good. Metabolic parameters have greatly improved at postoperative 1-month and 5-month (Supplementary Table 1). DKA was not diagnosed after surgery. Blood glucose levels as represented by the level of HbA1 and HbA1c, and the blood pressure returned to normal. The preoperative and postoperative BMI was 31.1 and 25.9, respectively. Routine histopathological examination and diagnostic evaluation suggested that “pituitary tumor in sellar region” was consistent with pituitary adenoma with extensive hemorrhagic infarction and secondary inflammatory fiber hyperplasia. Immunohistochemistry results were positive for GH (+), syn (+), CD56 (+), CGA (+), ER (focus+), cam 5.2 (+) and CK (Pan), and were negative for ACTH (-), FSH (-), TSH (-), LH (-), PRL (-), PR (-), Ki67 (1–2 %), and p53 (-). However, despite the well recovery, hypocortisol state and central hypothyroidism were observed after surgery. We treated the patient with cortisol 20 mg twice a day (morning and evening) and Euthyrox 25 ug once a day orally.

During treatment, active volume resuscitation and antacid-medicated stomach protection were noted. Further examination during the course of treatment revealed diabetic ketoacidosis, hyperlipidemia, hypocortical crisis (pituitary apoplexy, shock, ahyponatremia, low random total cortisol, and increase of production and secretion of ACTH), and acute kidney injury (based on KDIGO criteria and the condition that the serum creatinine level has raised from 54.4 umol/L on admission to 112 umol/L 16 h before transferred to EICU), as well as shortness of breath and decreased blood oxygen saturation. A large amount of hydrops was found in the abdominal cavity and pelvic cavity, which were treated once by plasma adsorption, and hydrocortisone 200 mg/D micropump maintenance treatment, insulin hypoglycemia, abdominal puncture drainage and other symptomatic support treatment. No obvious abnormalities in the patient’s diagnostic indicators were found during follow-up in the outpatient clinic for nearly half a year. The patient was contacted by the hospital via phone follow-up to the present. Long-term chronic use of thyroxine tablets and prednisone continued until the present, and the patient had normal blood glucose, slightly higher triglyceride levels (2.88 mmol/L), but no recurrence of pancreatitis.

## Discussion

The triad of DKA, HTG, and AP in a single patient has only rarely been reported in the literature [7]. In DKA, severe insulin deficiency can lead to disorders of glucose and lipid metabolism, which further leads to development of HTG and ultimately the resulting AP. When AP combines with DKA, it not only delays the diagnosis of AP due to the lack of specific symptoms and biomarkers, but also increases the consumption of hyperglycemia and vascular content. However, concurrent DKA has not been found to affect morbidity and mortality in patients with HTG-induced pancreatitis [8].

Growth hormone pituitary tumor apoplexy with DKA is also a relatively rare complication in adults [9]. Insulin insufficiency due to excessive growth hormone may be associated insulin resistance, glucose intolerance, and type 2 diabetes [10]. Impaired glucose and lipid homeostasis is the main feature of MetS [11]. Insulin resistance is considered to be a key factor in the development of MetS, and HTG is frequently observed in diabetic patients [12]. Patients with MetS, including diabetes, often manifest a typical lipid profile referred to as “diabetic dyslipidemia” [13].

Acute stress events, such as cerebrovascular accidents, infections and trauma, may add to the already disordered glucose and lipid metabolism in patients with MetS, directly inducing DKA [14]. During the course of DKA, insulin insufficiency activates the decomposition and release of fats from adipose tissue, leading to acceleration of very low-density lipoprotein (VLDL) synthesis in the liver, which is owed directly to elevated free fatty acid levels. In addition, the decreased lipoprotein lipase activity in peripheral tissues reduces removal of plasma VLDL, resulting in HTG [15], and moderate HTG is common during the onset of DKA [16], which induces direct toxicity in the pancreatic acinar cells and pancreatic capillaries. Meanwhile, HTG-induced pancreatitis may cause acute β-cell dysfunction, resulting in transient insulin deficiency and exacerbating ketoacidosis.

The triad of DKA, HTG, and AP may be associated with the level of HTG [17]. Severe HTG-related AP is an under-recognized disease [18], and hyperlipidemia appears to be the primary cause and phenomenon for AP attacks [19]. Studies that have examined the effect of previous diabetes on AP outcomes report that the risk of AP in diabetic patients is about 30 % higher than in those without diabetes [20]. DM and MetS, especially MetS including obesity and hyperlipidemia, are noted to have adverse effects on the course of AP, especially the impact on multiple organ systems, including the pancreas; in particular, severe HTG can lead to increased morbidity and mortality [5].

DKA is a rare manifestation of pituitary adenoma apoplexy. In our patient, the pituitary adenoma apoplexy seemed to directly induce DKA, creating a domino effect, which increases the already disordered metabolism of glucose and lipids in patients with MetS. The relative lack of insulin is accompanied by increased ketogenic activity in adipose tissue and glucagon in the liver. Moreover, the lack of insulin activates lipolysis and reduces VLDL in plasma during the course of DKA, and subsequent clearance then leads to HTG [15]. Hyperlipidemia is likely to be the main cause and phenomenon of AP, with risk of AP increased by high concentration of triglycerides [21]. Therefore, associations appear to exist between ketoacidosis, hyperlipidemia, and AP. Diabetes or impaired glucose tolerance are independent risk factors for AP secondary to HTG [22]. Patients with AP have higher risk of comorbid DKA and a history of diabetes, and serum triglyceride levels are significantly higher in these patients [23]. The long-term glyco- and lipotoxicities identified in our patient resulted in DKA, which is known to result from severe, but partially reversible, β-cell dysfunction. Classified as ketosis-ketodiabetes (KPD) syndrome, growth hormone control serves to maintain blood glucose at somewhat normal levels [24]. After the resection of the pituitary tumor, our patient was advised to stop insulin treatment, which was not only effective in regulating blood glucose levels, but also lowered triglyceride levels significantly (68.07 vs. 2.88 mmol/L).

DKA, HTG and AP can be seen with pituitary tumor in adult. Early recognition of these symptoms has important implications in the management of the patient as resection of the pituitary tumor, recovery duration, and prognosis can be altered. In patients presenting with ketoacidosis and elevated lipase levels, the treating physicians may perform serum amylase tests and early abdominal imaging in order to reach an early diagnosis of AP. In the presence of MetS and abnormally increased growth hormone levels, acute stress-induced metabolic disorders may be highly suspected. In such patients, thorough relevant examinations must be conducted, pituitary tumors and endocrine factors must be excluded, and the etiology must be clarified in order to manage diabetes and triglyceride levels effectively after tumor resection.

## Supplementary Information

**Additional file 1:**

## Data Availability

The data used to support the findings of this study are included within the article.

## Abbreviations

AP: Acute pancreatitis
CT: Computed tomography
DKA: Diabetic ketoacidosis
GH: Growth hormone
HTG: Hypertriglyceridemia
KPD: Ketosis-ketodiabetes
MetS: Metabolic syndrome
MRI: Magnetic resonance imaging
VLDL: Very low-density lipoprotein
WBC: White blood cell
