# Supplementary material for: Domino effect of pituitary growth hormone tumor complicated by diabetic ketoacidosis and pituitary apoplexy: a case report
Source: BMC Endocr Disord. 2021 May 26;21:109. doi: 10.1186/s12902-021-00768-9 (PMC8157700; doi:10.1186/s12902-021-00768-9)
Supplement: Supplementary file 1 — Additional file 1: [file 12902_2021_768_MOESM1_ESM.docx]

**Supplementary Table 1. Metabolic parameters before and after surgery.**

| **Blood test** | **On admission** | **Postoperative 1-month** | **Postoperative 5-month** | **Normal range** |
| --- | --- | --- | --- | --- |
| HbA1 | 17.60% | 8.80% | 7.30% | 6.30-9.00% |
| HbA1c | 15.10% | 7.10% | 5.60% | 3.60-6.00% |
| Total cholesterol | 21.64 mmol/L | 4.43 mmol/L | 3.86 mmol/L | 3.11-5.96 mmol/L |
| Triglycerides | 68.07 mmol/L | 1.27 mmol/L | 2.88 mmol/L | 0.34-1.70 mmol/L |
| High-density lipoprotein (HDL) cholesterol | 0.43 mmol/L | 1.15 mmol/L | 0.75 mmol/L | 1.04-2.05 mmol/L |
| Low-density lipoprotein (LDL) cholesterol | 1.22 mmol/L | 2.62 mmol/L | 1.64 mmol/L | 2.01-3.10 mmol/L |
| Non-HDL cholesterol | 21.21 mmol/L | 3.28 mmol/L | 3.11 mmol/L | 0.86-4.10 mmol/L |
| Apolipoprotein A1 | 0.67 g/L | 1.25 g/L | 0.91 g/L | 1.20-1.80 g/L |
| Apolipoprotein B | 0.28 g/L | 0.75 g/L | 0.67 g/L | 0.60-1.14 g/L |
| ApoB/ApoA | 0.42 | 0.60 | 0.74 | 0.40-1.10 |
| Free fatty acid | 1416.00 umol/L | 95.00 umol/L | 202.00 umol/L | 129.00-769.00 umol/L |
| Lactate dehydrogenase | 595.00 U/L | 145.00 U/L | 185.00 U/L | 120.00-250.00 U/L |
| Lipoprotein(a) | 38.00 mg/L | 137.00 mg/L | 94.00 mg/L | 0.00-300.00 mg/L |
